# Supplementary material for: Dynamically controlled Purcell enhancement of visible spontaneous emission in a gated plasmonic heterostructure
Source: Nat Commun. 2017 Nov 21;8:1631. doi: 10.1038/s41467-017-01870-0 (PMC5696373; doi:10.1038/s41467-017-01870-0)
Supplement: Supplementary file 1 — Supplementary Information [file 41467_2017_1870_MOESM1_ESM.pdf]

Supplementary Note 1. The proposed physical mechanism of modulation of optical response

We perform numerical electrostatic calculations (Device Lumerical, Lumerical Solutions, Inc.) to determine the carrier distribution in the top TiN electrode of the TiN/SiO_2/Ag heterostructure (Fig. 1) as a function of applied bias. This is achieved by numerically solving the Poisson and drift-diffusion equations (Supplementary Fig. 1). We assume that TiN has a bandgap of E_{bg} = 3.4 eV, electron affinity of χ = 8.3 eV, effective electron mass of m^* = 1.2 m_e. The DC permittivity and electron mobility of TiN are chosen as 9.3 and 1 $cm^2V^{-1}s^{-1}$, respectively.

The work functions of TiN films grown under different deposition conditions have been previously reported[1]. However, the carrier concentration of TiN films has not been specified in the cited work[1]. Since the carrier concentration of TiN strongly depends on fabrication conditions, it is not possible to accurately calculate the electron affinity of TiN by relying on the reported work function values. Since the cited paper discusses possibility of using TiN as a gate electrode, we assume that TiN used in that work is heavily doped. Hence, in the present study we assume that the work function of TiN with carrier concentration of 1.8×10^{22} cm^{-3} is 6 eV. In the device calculations, we used the mesh size of 0.05 nm.

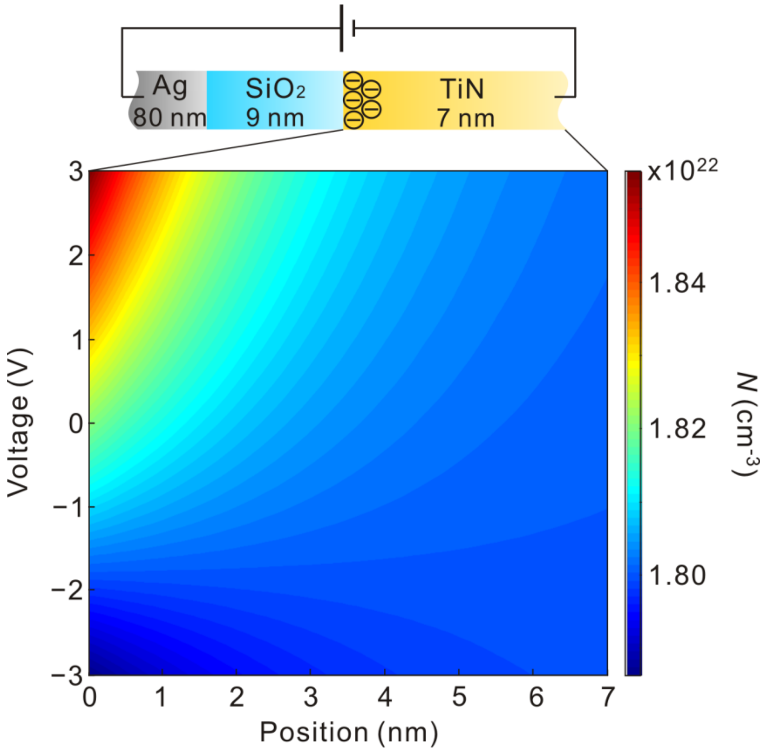

Supplementary Figure 1 |Simulated electron density in TiN as a function of position and applied bias. When we apply a positive bias, an electron accumulation layer is formed in TiN at the TiN/SiO_2 interface. At negative biases of –1.8 V and below, an electron depletion layer is formed in TiN at the TiN/SiO_2 interface. The optical modulation mechanism is based on a metal-oxide-semiconductor (MOS) field-effect dynamics. For the assumed value of electron affinity, at zero bias, we have an electron accumulation in the TiN.

Supplementary Note 2. Characterization of fabricated TiN films

For each TiN/SiO_2/Ag device, we fabricate additional large area control samples for performing Hall measurements and ellipsometry. The control samples consist of a Si substrate, followed by a 35 nm-thick SiO_2 layer, and an ultrathin TiN film. The carrier concentration (N) and electron mobility of each TiN film have been identified via Hall measurements (Supplementary Table 1).

N (cm^{-3})	5.9×10^{20}	2.6×10^{21}	1.8×10^{22}	4.1×10^{22}
Mobility (cm^{2}V^{-1}s^{-1})	0.558	0.35	0.059	5.8
Ellipsometry fitting model	2 Lorentz oscillators	1 Drude and 2 Lorentz oscillators	1 Drude and 2 Lorentz oscillators	1 Drude and 3 Lorentz oscillators
Optical property	Optically dielectric	Optically dielectric	ENZ	Optically plasmonic
Film thickness (nm)	8	34	7	46
Substrate	35 nm SiO_2/Si	35 nm SiO_2/Si	9 nm SiO_2/Ag	35 nm SiO_2/Si
Applied DC Power (W)/ voltage (V)	150/465	150/484	250/514	252/682
Ar/N_2 flow rate	5/0	5/0	5/0	5/0
Base Pressure (torr)	4.0×10^{-7}	4.0×10^{-7}	2.6×10^{-6}	3.1×10^{-6}
Gas pressure (mtorr)	5	5	3	3

Supplementary Table 1 | Material properties and growth parameters of TiN films. We deposit thin TiN films via DC sputtering. When sputtering TiN films we fix the Ar/N_2 flow rate, and the properties of the resulting films are controlled by changing DC power and voltage.

We measure the complex dielectric permittivity of the fabricated TiN films by using spectroscopic ellipsometry. We fit the ellipsometrically measured data by using the Drude-Lorentz model[2], which is a sum of a Drude term and two or three Lorentz oscillators:

$$\tilde{\varepsilon}_{\mathrm{TiN}}(\omega) = \varepsilon_{\infty} - \frac{\omega_p^2}{\omega^2 - i\Gamma\omega} + \sum_{j=1}^{3} \frac{f_j \times \omega_{oj}^2}{\omega_{oj}^2 - \omega^2 + i\gamma_j\omega}. \quad (1)$$

We identify the values of the free parameters incorporated in the model by fitting them to the ellipsometry data. There are three free parameters in the Drude terms of Supplementary Equation 1 including the damping factor Γ, plasma frequency ω_p, and ε_{∞}. The plasma frequency relates to the electron effective mass m^* and the carrier density of the film N as follows: $\omega_p = \sqrt{\frac{nq^2}{\varepsilon_0 m^*}}$. Here, q is the electron charge, ε_0 is the dielectric permittivity of vacuum. Each Lorentz oscillator in Supplementary Equation 1 contains three fitting parameters: the oscillator strength f_j, the damping factor γ_j, and the oscillator position ω_{oj}. Here, the index j numerates the Lorentz oscillators (j=1,2,3). Supplementary Table 2 summarizes the values of the obtained fitting parameters that have been used to produce Fig. 2.

N (cm^{-3})	E_p (eV)	Γ (eV)	f_1	E_{o1} (eV)	γ_1 (eV)	f_2	E_{o2} (eV)	γ_2 (eV)	f_3	E_{o3} (eV)	γ_3 (eV)	ε_{∞}
**5.9 × 10^{20}**	–	–	5.9	3.8	2.1	3.8	1.2	1.6	–	–	–	2.0
**2.6 × 10^{21}**	4.6	17.3	13.3	1.0	1.8	6.6	4.0	2.0	–	–	–	1.7
**1.8 × 10^{22}**	5.15	102	22	0.6	1.6	10	4.0	0.8	–	–	–	4
**4.1 × 10^{22}**	6.9	1.04	0.4	2.4	0.3	8.9	3.5	2.4	1.1	2.6	0.7	1.3

Supplementary Table 2 | The Drude-Lorentz fitting parameters for the complex dielectric permittivity of TiN. These values have been used to produce Fig. 2.

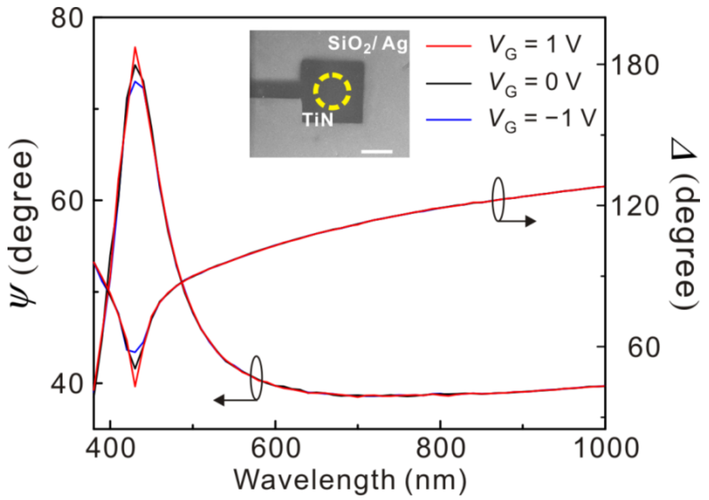

Supplementary Figure 2 | Reflectance modulation measured via focused beam spectroscopic ellipsometry. Δ and Ψ for the ENZ-TiN/SiO_2/Ag heterostructure as a function of wavelength at different voltages. In this measurement, the incidence angle is 60°. We observe modulation of Ψ and Δ under applied electrical bias. When electrical bias increases from −1 V to +1 V, we observe a monotonic increase of Ψ and monotonic decrease of Δ. The inset shows the SEM image of the device and the normal incident beam size of focused beam spectroscopic ellipsometry. The scale bar is 500 μm.

We also perform ellipsometry of our ENZ-TiN/SiO_2/Ag heterostructure under applied bias. However, the obtained data is not amenable to unambiguous interpretation due to the reasons described below. The area of our gate-tunable ENZ-TiN/SiO_2/Ag heterostructure is only 1 mm^2. The reason why we choose such small device area is that the thickness of our SiO_2 gate dielectric is only 9 nm. Larger area gate-tunable ENZ-TiN/SiO_2/Ag heterostructures would typically exhibit high leakage current upon biasing due to defects of gate dielectric. When performing ellipsometry it is important that the incoming light is reflected form the working area of the device only. Hence, during ellipsometry measurements we have to use the focused light beam. However, the beam spot is still larger than the working area of the device, especially for larger incidence angles. By using this approach we obtain Ψ and Δ for different incidence angles. Ψ and Δ are defined by the following formula: $r_p/r_s=\tan(\Psi)e^{i\Delta}$, where r_p and r_s are the reflection coefficients of the p- and s-polarized light, respectively. However, we are not able to obtain a reliable ellipsometry fit to extract dielectric permittivity of TiN. Nevertheless, we have acquired Ψ and Δ for a given incidence angle (60°) and different applied biases (Supplementary Fig. 2). We observe optical modulation around 430 nm where Ψ and Δ exhibit maximum and minimum, respectively. Finally, we would like to note that since the ellipsometry is performed

several months after fabricating the sample, the observed optical modulation is modest as compared to the result shown in Fig. 3 of the manuscript.

As we have previously mentioned, because of the small device area, we are not able to obtain reliable ellipsometry fits for the ENZ-TiN, which is incorporated in the ENZ-TiN/SiO_2/Ag heterostructure. We identify the dielectric permittivity of the TiN in the TiN/SiO_2/Ag heterostructure by fitting the parameters of the Drude-Lorenz model to the reflectance spectrum. The reflectance spectrum taken under normal incidence with 5X objective (Olympus, with numerical aperture of 0.14), which focuses a supercontinuum Fianium laser down to a small spot of 3 μm in diameter. This spot size is much smaller than the working area of our device, which is 1 mm^2. When fitting the parameters of the Drude-Lorenz model to the reflectance data, we use the electron mobility and carrier concentration values derived via Hall measurements (Supplementary Table 1). As seen in Supplementary Fig. 3, the calculated and measured reflectance are in good agreement. As demonstrated in Fig. 2, the real part of the dielectric permittivity of our TiN shows double-ENZ crossing that is in good agreement with recent literature reports [3].

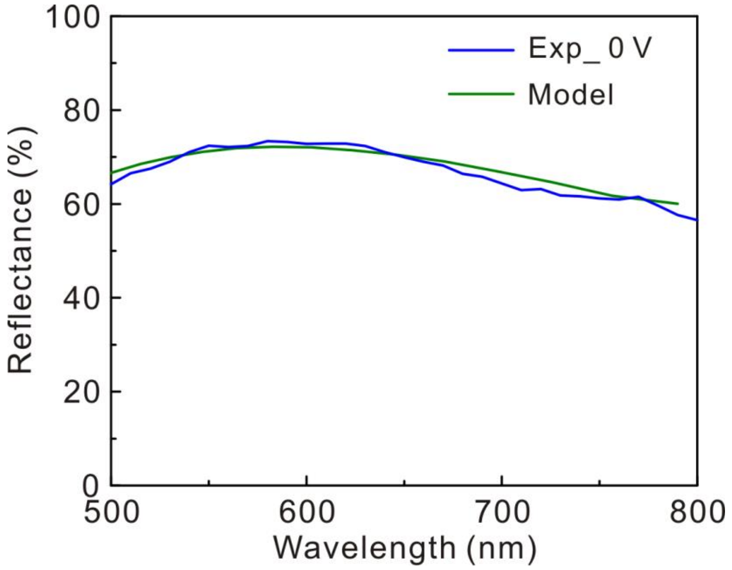

Supplementary Figure 3 | Measured and calculated reflectance of the epsilon-near-zero (ENZ)-TiN/SiO_2/Ag heterostructure. The dielectric permittivity of the ENZ-TiN is extracted from reflectance measurements.

When analyzing the ellipsometry data, we use two or three Lorentz oscillators to fit the data obtained from the fabricated films. The key issue here is that optical properties of TiN films strongly depend on the film stoichiometry, that is the ratio of titanium and nitrogen, impurities, such as residual oxygen or oxygen introduced due to post growth oxidation, grain size, and density/porosity [2]. The grain size affects the mean free path of the conduction electrons, while the density/porosity influences the conduction electron density. All these factors likely differ from one film to another resulting in different values of fitting parameters of a Drude-Lorentz model, such as electron mobility and frequencies of Lorentz oscillators. In particular, it has been previously shown that the spectral position of the zero crossing (ENZ point) is an indicator of film stoichiometry. It has been shown that for stoichiometric TiN films this crossing occurs at 2.65 *e*V (λ=468 nm) [4]. As seen in Fig. 2a, none of our films has an ENZ crossing at λ= 468 nm. Hence, all the films reported in this work are non-stoichiometric. Moreover, it has been recently shown that amount of residual oxygen in the sputtering chamber can dramatically affect optical properties of TiN films [3,5,6]. Based on these reports, we expect that each fabricated film has different chemical and structural composition. The review article [2] discusses the relation between the band structure of TiN obtained via density functional theory (DFT) calculations and the spectral positions of Lorentz oscillators in an ellipsometry fit. As seen in Supplementary Table 2 of the mentioned review article [2], the spectral positions of Lorentz oscillators show significant variation. Moreover, the reported spectral position values do not always agree with the DFT calculations. The reason for this is that the DFT calculations are performed for stoichiometric TiN, while the films shown in our work as well as a number of films shown in the review article [2] are non-stoichiometric. Moreover, we perform compositional analyses on 135 nm-thick TiN films deposited on Si substrates via DC sputtering. The sputtering conditions are chosen to be identical to those used to deposit TiN in the tunable ENZ-TiN/SiO_2/Ag heterostructure. We find that the stoichiometry of our film is given as $TiN_{0.8}O_{0.2}$, where oxygen has been introduced into our film unintentionally. This result is consistent with previous reports [5,6]. Hence, most likely, 7 nm-thick TiN films incorporated in our TiN/SiO_2/Ag films also include a significant amount of oxygen impurities. Based on this, it is not straightforward to relate positions of Lorentz oscillators to the band structure of our TiN, due to limited knowledge of the band structure of the actual film. To summarize, the

choice of the model that we use to fit the ellipsometry data (two versus three Lorentz oscillators, etc.) is largely dictated by the composition and structure of the films, which varies. Due to a limited knowledge of the dependence of the TiN band structure on the material stoichiometry and the amount of incorporated oxygen impurities, we are not able to perform a direct comparison between the positions of Lorentz oscillators and the material band structure.

One can obtain indirect evidence of compositional and structural differences in sputtered TiN films when analyzing the dependence of the imaginary part of the measured dielectric permittivity Im(ε) on the carrier concentration of the films. Indeed, Im(ε) monotonically increases with the carrier concentration for carrier concentrations less than or equal to 1.8×10^{22} cm^{-3} and abruptly decreases for the sample with the carrier concentration of 1.8×10^{22} cm^{-3}. This kind of behavior has been previously reported in literature [5]. In the mentioned paper, Im(ε) of the "intermediately doped" TiN is greater than Im(ε) of the "dielectric" TiN [5]. On the other hand, over a broad wavelength range, Im(ε) of the "metallic" TiN is smaller than Im(ε) of the "intermediately doped" or "dielectric" TiN [5]. It is argued that when the oxygen impurity concentration in TiN is lessened, the crystallinity of TiN films is improved. As a result, Re(ε) becomes more negative, and Im(ε) decreases.

Interestingly, in the visible wavelength range we observe a non-monotonic behavior of Im(ε) as a function of wavelength (Fig. 2b) that is consistent with the previously reported functional dependences of Im(ε) on wavelength [2,3,5]. On the other hand, as we observe in our work, Im(ε) typically grows monotonically as a function of wavelength in the near-infrared wavelength range [2,3,5].

Supplementary Note 3. Effect of carrier concentration of TiN on reflectance of TiN/SiO_2/Ag heterostructures

In each of the fabricated TiN/SiO_2/Ag heterostructures there are QDs embedded in the SiO_2 layer. Hence, one might assume that the observed optical modulation is due to the variation of the effective refractive index of InP QDs embedded in the structure. However, this is not a possible mechanism since only heterostructures with specific carrier concentration of TiN yield reflectance modulation under applied bias (Supplementary Fig. 4). This indicates that the presence of TiN film is crucial for observation of optical modulation.

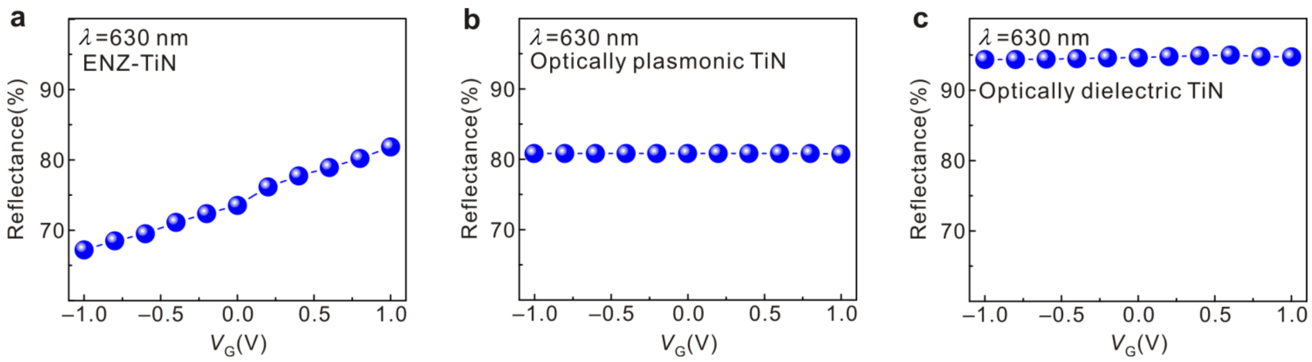

Supplementary Figure 4 | Reflectance of TiN/SiO_2/Ag heterostructures as a function of applied bias at a quantum dot emission wavelength of λ=630 nm. The fabricated TiN film has different carrier concentration for each measured heterostructure. (**a**) Reflectance modulation under applied bias when the TiN is in the epsilon-near-zero (ENZ) phase ($N = 1.8 \times 10^{22}$ cm^{-3}). We observe no detectable reflectance modulation when TiN is in (**b**) optically plasmonic ($N = 4.1 \times 10^{22}$ cm^{-3}) or (**c**) optically dielectric phase ($N = 2.3 \times 10^{20}$ cm^{-3}).

Supplementary Note 4. Modulation speed of optical response of TiN/SiO_2/Ag heterostructures

We perform the modulation frequency measurements four months after the sample was fabricated. As a result, by the time of the modulation frequency measurements, our sample has already been degraded. Supplementary Fig. 5 shows the reflectance spectrum of the degraded sample taken right before the modulation frequency measurements. Degraded samples typically exhibit an increased contact resistance between the TiN and contact electrode. Therefore, we believe, we need to apply a larger electrical bias of ±5 V to observe reflectance modulation. We would like to point out that the application of higher voltages does not necessary imply that the built-in electric field inside the heterostructure is going to be larger. Moreover, the reflectance characteristics of the degraded sample have also been modified; likely, due to sample oxidation (compare Fig. 3a and Supplementary Fig. 5). Despite sample degradation, the sample still exhibits optical modulation under applied bias. We perform reflectance modulation measurements using the degraded sample. Importantly, we observe a modulation frequency of 20 MHz, and the detected reflectance values show a perfect match with the reflectance values measured in Supplementary Fig. 5. Finally, we would like to note that the samples can be protected by depositing a thin encapsulation layer, such as Al_2O_3.

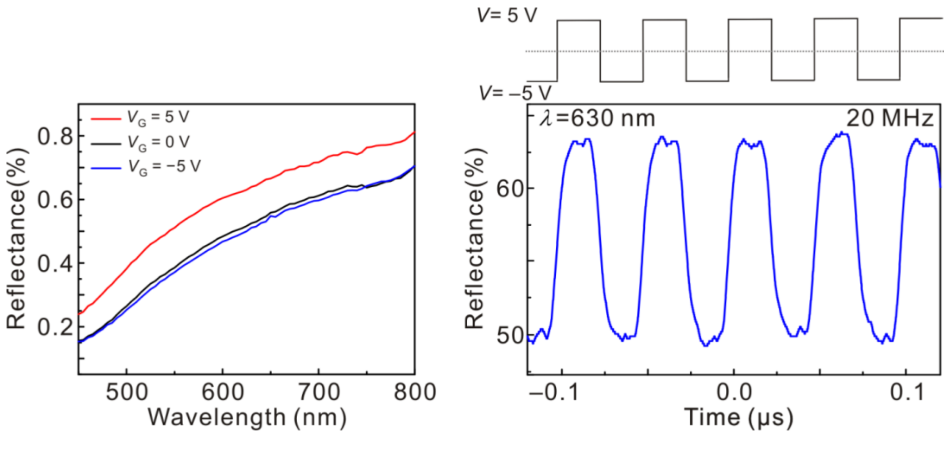

Supplementary Figure 5 | Modulation sped of TiN/SiO_2/Ag heterostructure. We measure the reflectance of the TiN/SiO_2/Ag heterostructure under modulating bias that changes between –5 V and 5 V with a modulation frequency of 20 MHz. The modulation amplitude varies between 50% and 63%. Our detection frequency was limited by the Si photodetector response time. This implies that the modulation frequency of our device could potentially be higher than 20 MHz.

Supplementary Note 5. Calculation of reflectance under applied bias

As it has been shown in Supplementary Note 3, meeting ENZ condition in the TiN film is crucial for observation of optical modulation. The measured real and imaginary parts of the dielectric permittivity of TiN are shown in Fig. 2. Although when we increase the TiN carrier concentration, TiN undergoes transition from optically dielectric to optically plasmonic phase, the imaginary part of its dielectric permittivity does not increase in a monotonic fashion (Fig. 2b). This indicates that to accurately model the bias-dependent optical response of the TiN film in the TiN/SiO_2/Ag heterostructure, one cannot simply change the carrier concentration in the Drude-Lorentz model while keeping intact the parameters of the Lorentz oscillators defined at zero bias. Therefore, in our simulations we describe the accumulation layer of TiN by the experimentally measured dielectric permittivity of the heavily doped TiN film.

Next, we calculate the reflectance of the TiN/SiO_2/Ag heterostructure. In our simulations, we assume layer thicknesses identical to those identified from the transmission electron microscopy image (Fig. 1). The calculated TiN/SiO_2/Ag heterostructure consists of 80 nm-thick Ag back reflector, 9 nm-thick SiO_2, and 7 nm-thick TiN. We assume that upon application of electrical bias, a 1 nm-thick modulated TiN layer is formed in TiN at TiN/SiO_2 interface. For example, when applying a positive bias a 1 nm-thick charge accumulation layer is formed in TiN at TiN/SiO_2 interface. As a result, the modulated layer becomes optically plasmonic and the reflectance of the heterostructure is increased (Supplementary Fig. 6). On the other hand, under negative bias, the permittivity of the modulated TiN layer moves toward the ENZ region that results in the suppression of the reflectance. Finally, we note that in our simulations the mesh size is 2 nm in the directions along the layers of TiN/SiO_2/Ag heterostructure, while for the direction normal to the layers the mesh size is 0.5 nm.

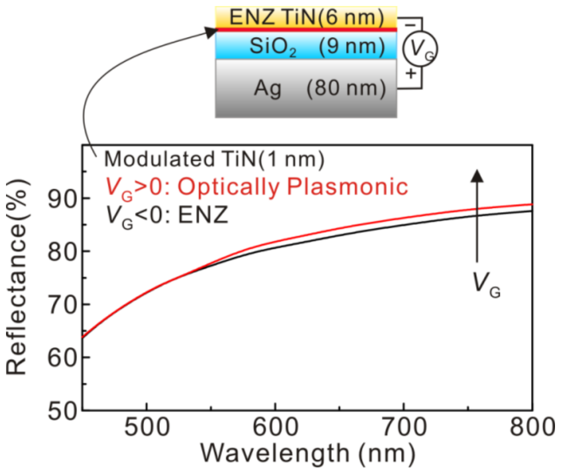

Supplementary Figure 6 | Calculated reflectance modulation of TiN/SiO_2/Ag heterostructure. Calculated reflectance spectrum of the TiN/SiO_2/Ag heterostructure when the TiN is in the epsilon-near-zero (ENZ) region (N=1.8 × 10^{22} cm^{-3}). The reflectance increases when we apply a positive bias. The dielectric permittivity of the TiN films is shown in Fig. 2.

Our calculations show that the observed reflectance modulation cannot be fully explained by simply changing the carrier concentration in the Drude term of the Drude-Lorentz model that defines the dielectric permittivity of TiN. The carrier concentration of our ENZ-TiN film (N=1.8 × 10^{22} cm^{-3}) is at least 2 orders of magnitude higher as compared to the carrier concentrations that one typically deals with in ITO-based gate-tunable devices [7] (N=3 × 10^{20} cm^{-3}). Therefore, a number of different effects may contribute to the observed optical modulation. One possible effect is the electron effective mass's dependence on applied voltage due to nonparabolicity of conduction band. Another possible contributor is the dependence of the electron mobility on applied bias. In Supplementary Fig. 6 we have assumed that the dielectric permittivity of 1 nm thick accumulation layer in TiN is given by the measured dielectric permittivity of TiN film with carrier concentration of N= 4.1 × 10^{22} cm^{-3} (Fig. 2). This assumption is, strictly speaking, not correct; however, it allows us to estimate the sensitivity of the calculated reflectance with respect to the variation of the refractive index of the 1 nm-thick TiN layer, which is located at the interface of TiN and SiO_2 (Supplementary Fig. 6). To summarize, due to high carrier concentration of TiN films, modifying carrier concentration in the Drude term of the Drude-Lorentz model cannot reproduce experimentally observed reflectance modulation.

Supplementary Note 6. Modulation of PL intensity of InP QDs embedded in active/passive heterostructures

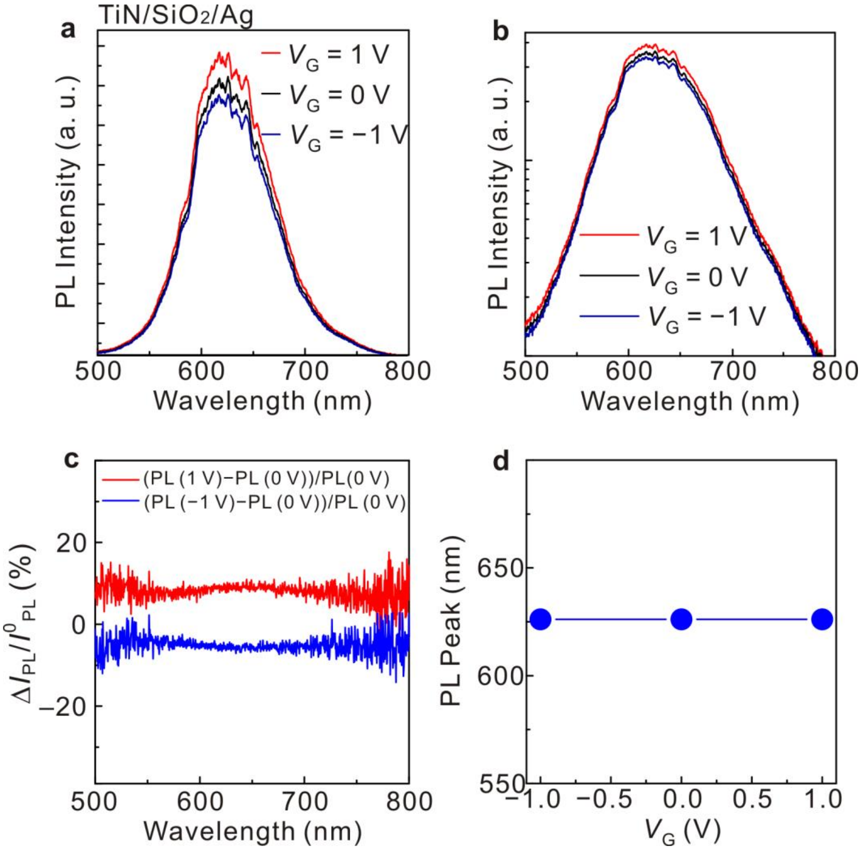

Supplementary Figure 7 | Modulation of photoluminescence (PL) intensity of InP quantum dots embedded in the gated TiN/SiO_2/Ag active plasmonic heterostructure. (a) PL intensity spectra for different gate voltages in a linear scale (the same PL spectra are shown in Fig. 4a). **(b)** PL spectra in a log scale. **(c)** Differential PL spectra. Red line: (PL(1V)–PL(0V))/PL(0V) and blue line: (PL(–1V)–PL(0V))/PL(0V). **(d)** PL peak position as a function of applied voltage. We observe no detectable wavelength shift.

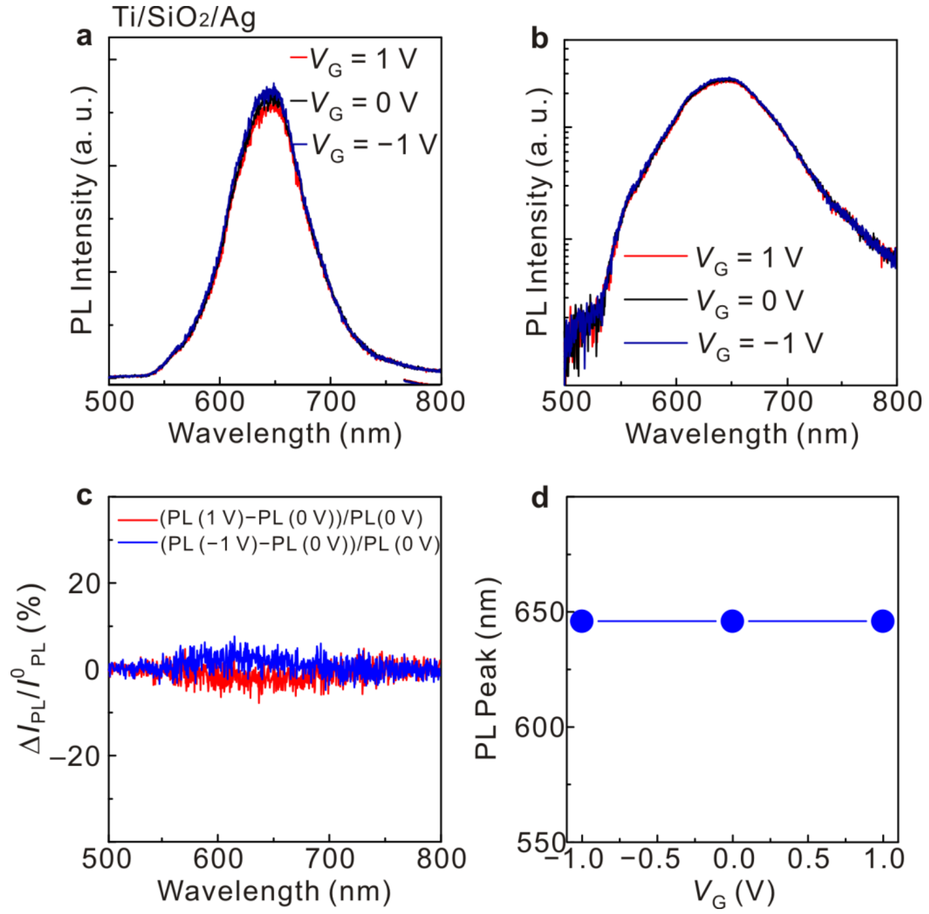

Supplementary Figure 8 | Modulation of the photoluminescence (PL) intensity of InP quantum dots embedded in the gated Ti/SiO_2/Ag passive heterostructure. (a) PL intensity spectra for different gate voltages in a linear scale (the same PL spectra are shown in Fig. 4b). **(b)** PL spectra in a log scale. **(c)** Differential PL spectra. Red line: (PL(1V)–PL(0V))/PL(0V) and blue line: (PL(–1V)–PL(0V))/PL(0V). **(d)** PL peak position as a function of applied voltage. We observe no detectable wavelength shift.

Supplementary Note 7. Calculation of local density of optical states (LDOS)

We calculate the local density of optical states (LDOS) in the SiO_2 gap of the TiN/SiO_2/Ag heterostructure via finite difference time domain (FDTD) method. In our calculations we model an InP/ZnS QD as a point dipole source located in the lossless sphere with a diameter of 4.5 nm and refractive index of 3.5. In our calculations we assume that the QD is located in the center of the SiO_2 layer, and the direction of the dipole moment is normal to the layers of the heterostructure. The refractive index of SiO_2 is assumed to be n_{SiO2}= 1.47. The optical permittivity of Ag and TiN are determined by ellipsometry measurements. It is known that the LDOS is proportional to the power radiated by a point dipole in the given environment[8]. Thus, we use FDTD to calculate LDOS enhancement with respect to LDOS in a bulk lossless dielectric with refractive index of 3.5 (Fig. 4d). It is worth mentioning that while calculating the LDOS, one should average out over different orientations of the dipole moment.

To enhance the amount of observed LDOS modulation, we suggest using a plasmonic cavity (Supplementary Fig. 9a) that can be formed by placing an appropriately designed patch antenna on top of an Ag/SiO_2/TiN heterostructure (akin to previously demonstrated reflectarray antennas [9,10]). It has been shown that the lifetime of a quantum emitter embedded in the dielectric gap of the patch antenna resonator is very sensitive to gap thickness[10]. If the resonant wavelength of the cavity is aligned with the emission wavelength of the emitter, this configuration will yield a large modulation of the PL intensity under an applied bias. Supplementary Fig. 9b shows the distribution of the electric field excited by the point dipole embedded in the 5 nm thick gap of the proposed patch antenna structure (Supplementary Fig. 9a). The assumed dielectric permittivity of 7 nm thick TiN film is identical to that of ENZ-TiN film used in the present work (the film with carrier concentration N= 1.8 $\times 10^{22}$ cm^{-3} from Fig. 2). In the case of a positive bias, we assume that a 1 nm thick accumulation layer is formed in TiN at the TiN/SiO_2 interface. The dielectric permittivity of the accumulation layer is assumed to be identical to that of the metallic TiN described in the present work (the film with carrier concentration N= 4.1 $\times$ 10^{22} cm^{-3} from Fig. 2). The LDOS enhancement for two different biases is shown in Supplementary Fig. 9c. As demonstrated in Supplementary Fig. 9, the suggested structure enables significant LDOS modulation under an applied bias. The simulation shown in Supplementary Fig. 9

aims to demonstrate the sensitivity of the lifetime of the emitter with respect to variation of the refractive index of the material in the gap, despite the difference in dielectric permittivity between the value assumed for the TiN accumulation layer and that of metallic TiN derived in the present work (N= 4.1 × 10^{22} cm^{-3}). Finally, we would like to note that using a patch antenna resonator can increase the emission intensity from the device by two orders of magnitude[9].

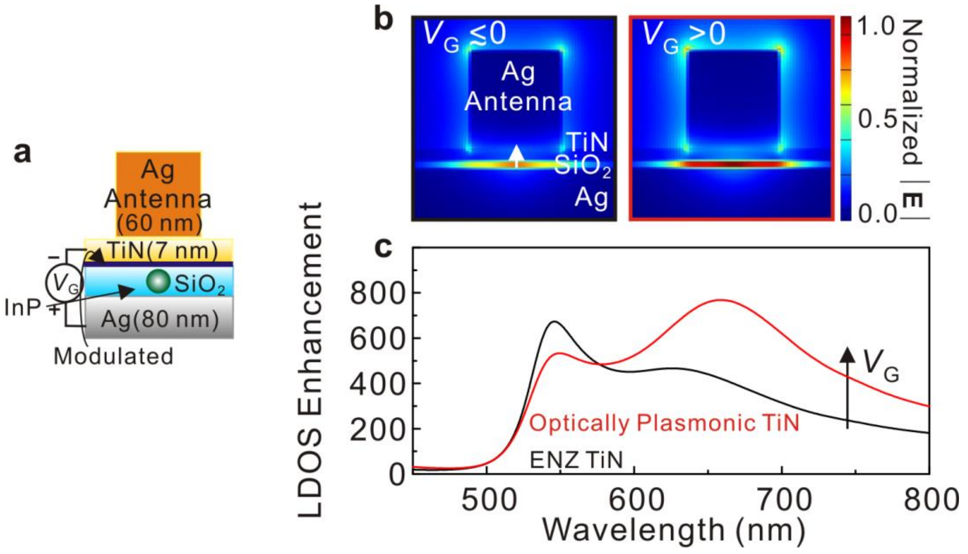

Supplementary Figure 9 | (a) Schematic of the proposed patch antenna with a quantum emitter embedded in a 5 nm-thick SiO_2 layer. **(b)** Calculated electric field distribution in the patch antenna structure. The depicted electric field is excited by the point dipole oriented normally to the planar layers of Ag, SiO_2, and TiN. **(c)** Calculated z-projection of the local density of optical states (LDOS) enhancement with respect to the value of the LDOS in the bulk InP, as a function of the emission wavelength, for zero bias and a positive bias.

Supplementary Note 8. Lifetime of InP/ZnS core-shell quantum dots on a bare Si substrate

The lifetime of QDs on a bare Si substrate is 11 ns (Supplementary Fig. 10). This shows that embedding QDs into the TiN/SiO_2/Ag plasmonic heterostructure results in 28-fold reduction of the QD lifetime as compared to the lifetime of QDs on bare Si.

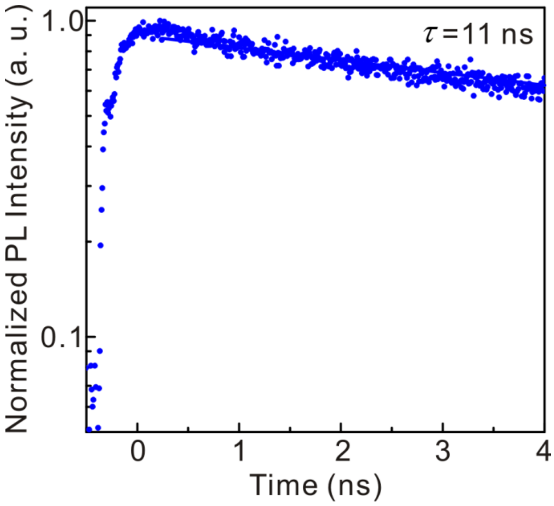

Supplementary Figure 10 | Lifetime of InP/ZnS core-shell quantum dots (QDs) on a bare silicon substrate. The measured PL lifetime of InP QDs is 11 ns.

Supplementary Note 9. Spatial distribution of optical frequency electric field in TiN/SiO_2/Ag heterostructures

Supplementary Fig. 11 shows the simulated spatial distribution of the optical frequency electric field |**E**| radiated by a point dipole source (λ=630 nm). We calculate the optical frequency electric field 30 nm away from the point dipole source. When the point dipole is located in the SiO_2 spacer, we observe considerable field enhancement at the SiO_2/TiN interface (Supplementary Fig. 11a). On the other hand, when the point dipole is located on top of the TiN layer, the optical frequency electric field emitted by the dipole does not completely overlap with the modulated TiN layer (Supplementary Fig. 11b). As a result, no spontaneous emission modulation under applied electrical bias is observed. Finally, we note that the observed modulation depth can be increased if we replace SiO_2 by a gate dielectric with higher DC permittivity such as HfO_2 or Al_2O_3.

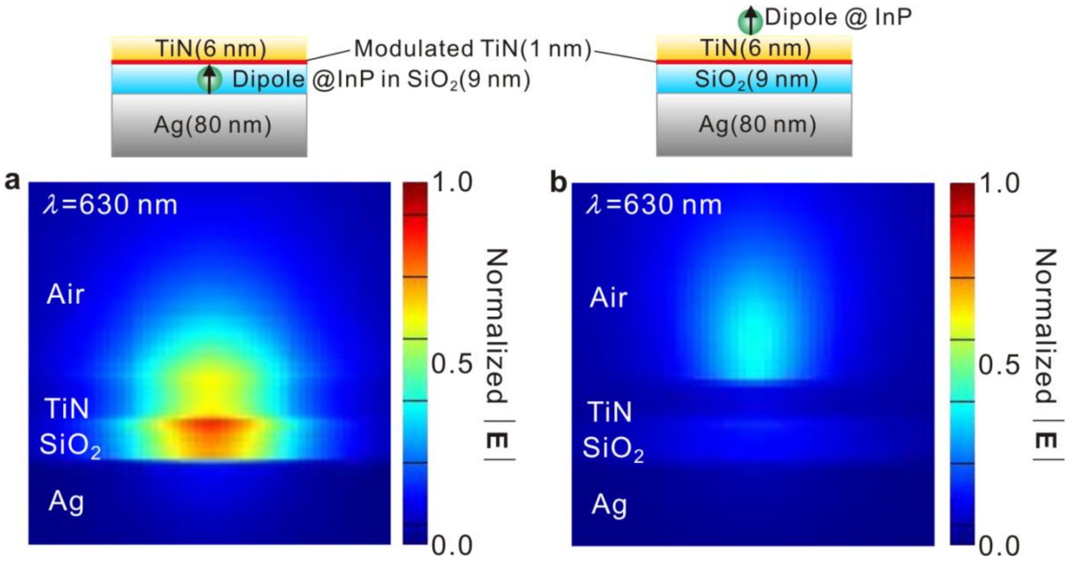

Supplementary Figure 11 | Spatial distribution of optical frequency electric field |E| (λ=630 nm). (**a**) Optical frequency electric field |**E**| radiated by a quantum dot (QD) embedded in the SiO_2 layer is tightly confined within that layer. (**b**) When a QD is placed on top of the TiN layer, the optical field in the active TiN layer is quite weak. As a result, in case (**b**) no detectable local density of optical states (LDOS) modulation is observed. This indicates that high optical field intensity |**E**| in the nearby active TiN layer is crucial for observation of LDOS modulation.

Supplementary Note 10. Quantum yield of InP QDs embedded in TiN/SiO_2/Ag heterostructures

We measure quantum yield of InP QDs embedded in the SiO_2 dielectric layer of the TiN/SiO_2/Ag heterostructure by referencing it to the quantum yield of standard florescence dye (Rhodamine 6G). Quantum yield of Rhodamine 6G is known to be 95% [11]. To determine quantum yield of our InP QDs embedded in SiO_2 dielectric layer (QE), we use the following formula:

$$\mathrm{QE}=\mathrm{QE}_{\mathrm{R6G}}\ (I/I_{\mathrm{R6G}})(A_{\mathrm{R6G}}/A)(n_{\mathrm{host}}/n_{\mathrm{R6G}})^2\ . \quad (2)$$

Here I and I_{R6G} denote the integrated PL intensity of our InP QDs embedded in SiO_2 dielectric layer and Rhodamine 6G, respectively, while A and A_{R6G} denote absorbance in InP QD embedded in SiO_2 dielectric layer and Rhodamine 6G dyes, respectively. Absorbance is measured at excitation wavelength of 488 nm. Effective refractive index of the QD host is n_{host}= 1.63, while Rhodamine dyes are suspended in ethanol with refractive index n_{R6G}=1.36. The quantum yield of Rhodamine 6G dye is known to be QE_{R6G} =95%. We measure reflectance (R) and transmitance (T) spectra under normal incidence by using a home-built integrating sphere setup with supercontinuum laser source (Fianium laser). To achieve near Lambertian reflectivity, we have conformally covered the integrating sphere with $BaSO_4$. While the photodetector in the sphere is recording reflectance and transmittance spectra, the intensity of the incident beam is simultaneously measured by a reference Si photodiode. Finally, the absorption spectrum (A) is obtained by using the relation A=1–R–T (Supplementary Fig. 12). Furthermore, we used the standard light source (Thorlabs Stabilized Tungsten-Halogen Light Source: SLS201L) to calibrate the spectral response for the PL emission. As a result, when no electrical bias is applied, the measured quantum yield of the InP QDs embedded in a 9 nm-thick SiO_2 layer is 15% .

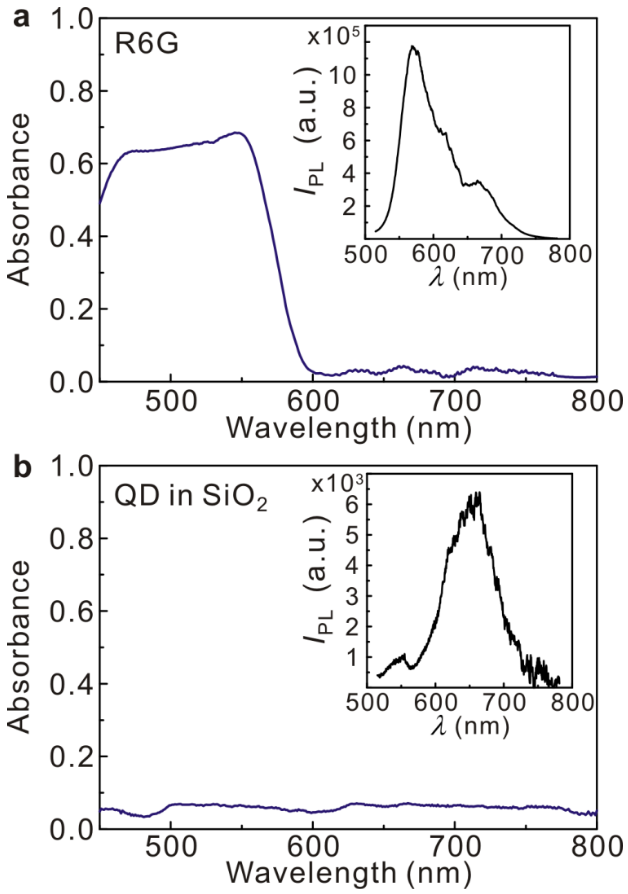

Supplementary Figure 12 | Quantum yield of InP quantum dots (QDs) embedded in SiO_2. (**a**) Absorbance spectrum of the Rhodamine 6G dye molecules suspended in ethanol. Inset shows the photoluminescence spectrum of the Rhodamine 6G dye molecules. (**b**) Absorbance spectrum of InP QDs (9% fill fraction) embedded in SiO_2 layer. Inset shows photoluminescence spectrum of InP QDs embedded in SiO_2. The measured quantum yield of InP QDs is 15%.

Supplementary Note 11. Calculation of radiative emission decay rate

We perform time-resolved PL measurements to identify the total decay of InP QDs at zero bias Γ_{tot} $=2.56 \times 10^9$ s^{-1}. We assume that the quantum yield of QDs embedded in heterostructure is 15%, the calculated radiative decay rate of QDs at zero bias is $\Gamma^0_{rad}=0.38\times10^9$ s^{-1}. Since InP QDs are embedded in the SiO_2 layer of the TiN/SiO_2/Ag heterostructure, when calculating radiative decay rates, we take into account absorption in the top TiN layer in order to have effective excitation rate. Hence, the bias dependent radiative emission decay rate (Γ_{rad}), is given by the following formula [10,12]:

$$\Gamma_{rad}(V_G)/\ \Gamma_{rad}{}^0 = (I_{PL}(V_G)/I_{PL}{}^0)[(1-A_{laser}(V_G))/(1-A_{laser}{}^0)]\ . \tag{3}$$

I_{PL} is the voltage-dependent PL peak intensity, and A_{laser} stands for absorbance of the TiN film at the excitation wavelength (Supplementary Figs. 13 and 14). V_G denotes the gate voltage applied between the Ag and TiN layers in the TiN/SiO_2/Ag heterostructure.

We obtain that the gate-tunable radiative decay rate changes when the applied gate voltage ranges between −1 V and +1 V. The quantum yield of an emitter η is defined as a ratio of radiative and total decay rates $\eta=\Gamma_{rad}/\Gamma_{tot}$. Thus, we identify that the quantum yield of InP QDs embedded in the TiN/SiO_2/Ag plasmonic heterostructure varies when the gate voltage V_G is increased from −1 V to +1 V. This *in situ* control of quantum yield is a unique consequence of the bias-induced modulation of LDOS.

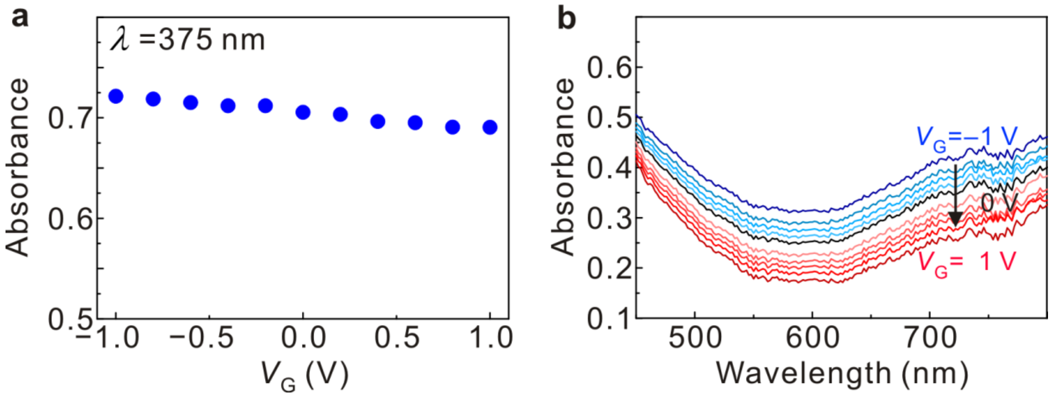

Supplementary Figure 13 | Absorbance of epsilon-near-zero (ENZ)-TiN/SiO_2/Ag plasmonic heterostructure. (**a**) Measured absorbance as a function of applied bias at the laser excitation wavelength of λ=375 nm. (**b**) Measured absorbance spectrum of the ENZ-TiN/SiO_2/Ag plasmonic heterostructure for different applied biases. Our calculations show that the observed absorption primarily occurs in the top TiN layer.

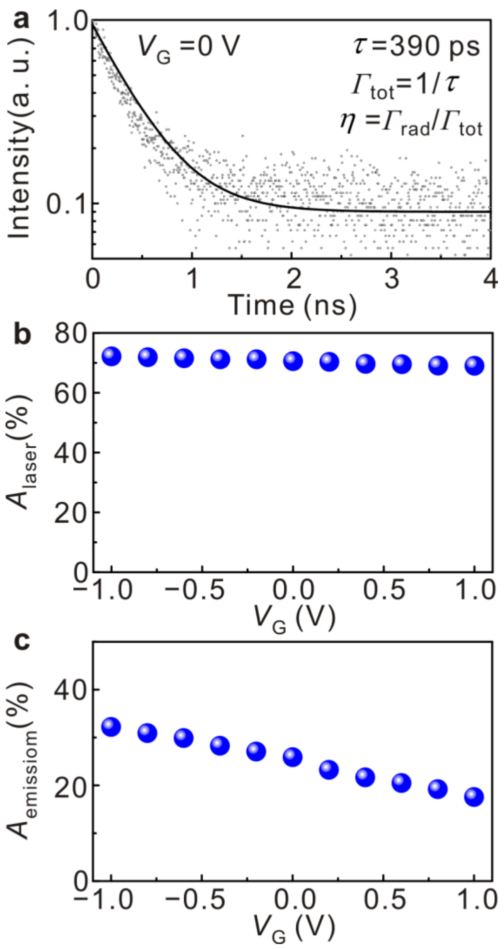

Supplementary Figure 14 | Lifetime at zero bias, and absorbance at excitation and emission wavelengths. (a) By using time-resolved photoluminescence measurements, we identify the total decay rate of the InP quantum dots at zero bias Γ_{tot} =2.56 × 10^9 s^{-1}. **(b)** Absorbance at the excitation wavelength (λ=375 nm) in the epsilon-near-zero (ENZ)-TiN/SiO_2/Ag heterostructure. **(c)** Absorbance at the emission wavelength, λ=630 nm, in the ENZ-TiN/SiO_2/Ag heterostructure. The Absorbtion primarily occurs in the top TiN layer.

Supplementary Note 12. Dependence of the PL intensity on excitation power

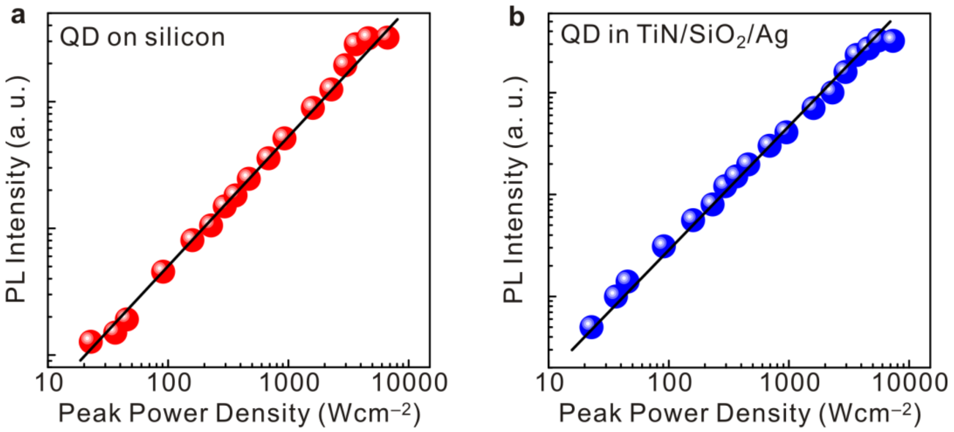

Supplementary Figure 15 | Dependence of the photoluminescence (PL) intensity on excitation power. (**a**) PL intensity of the InP quantum dots (QDs) directly placed on a silicon substrate as a function of excitation power. The observed unity slope of the curve is a characteristic feature of the spontaneous emission (SE) process. (**b**) PL intensity of the InP QDs embedded in the TiN/SiO_2/Ag plasmonic heterostructure is linearly proportional to the excitation power. In the present work, the local density of optical states (LDOS) modulation is measured at a pump power density of 7.5 kWcm^{-2}.

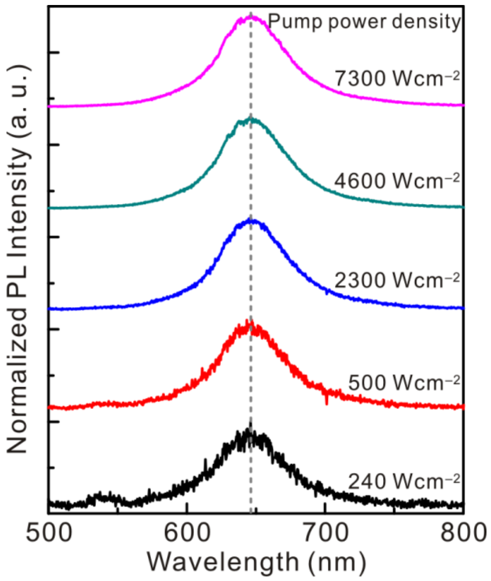

Supplementary Figure 16 | Normalized photoluminescence (PL) spectra for different excitation powers. PL spectra of InP quantum dots embedded in a TiN/SiO_2/Ag plasmonic heterostructure. To facilitate the comparison between different PL spectra, we normalize the recorded PL spectra so that they have identical amplitude.

Supplementary Note 13. Elimination of the hypothesis of optical modulation due to ionic conductance

In this particular TiN/SiO_2/Ag gated heterostructure, electrical breakdown occurs at an applied voltage of 10 V. (Supplementary Fig. 17a). We perform cyclic voltammetry measurements, to verify that in our structure there is no migration of silver ions that could possibly contribute to the observed optical modulation. Our cyclic voltammetry measurements show no hysteresis. This indicates that there is no ion migration in our system. The observed reflectance modulation speed of 20 MHz also conflicts with the hypothesis that the ionic migration could play a role in observed optical modulation. Indeed, reported optical modulation frequencies due to Ag or Au ion migration are smaller than 1 KHz[13-15].

Next, we perform a final control experiment to confirm that the observed modulation of optical response of the TiN/SiO_2/Ag heterostructure is due to charge accumulation or depletion in the TiN layer. Thus, we fabricate an indium tin oxide (ITO)/SiO_2/Ag planar heterostructure consisting of a 10 nm-thick ITO layer, 9 nm-thick layer of SiO_2, and an optically thick Ag back reflector. No significant reflectance modulation is observed at applied electrical bias rangimg between – 10 V and +10 V (Supplementary Fig. 18). The carrier concentration of the deposited ITO film is 3×10^{20} cm^{-3}, and the ENZ wavelength of the ITO film is close to the telecom wavelength. As a result, bias-induced transition from optically insulating to optically metallic phase occurs around telecom wavelength and not in the visible spectral range. Since this transition is crucial for observation of optical modulation, the ITO/SiO_2/Ag gated heterostructure shows no optical modulation in the visible wavelength range.

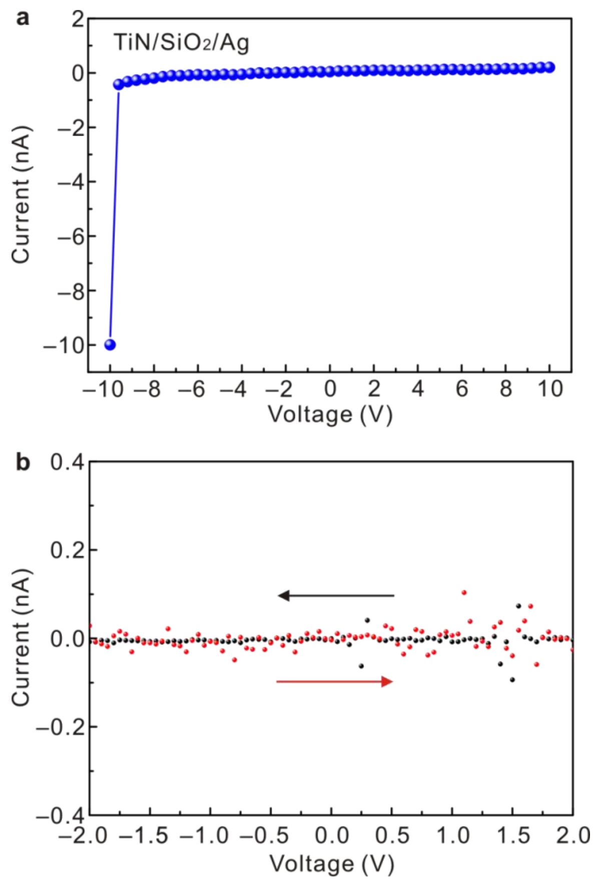

Supplementary Figure 17| *I-V* measurements for a TiN/SiO_2/Ag plasmonic heterostructure. (**a**) *I-V* curve indicates that the breakdown voltage is equal to 10 V. This corresponds to a breakdown field of 11 $MVcm^{-1}$. (**b**) Cyclic voltammetry measurements show no hysteresis (the arrows indicate direction of the voltage sweep starting at zero). In addition, the measured currents were in a sub-pico-Ampere range, indicating that SiO_2 is a good insulator. This piece of evidence suggests that in our system there is no ion migration that could possibly give rise to observed optical modulation.

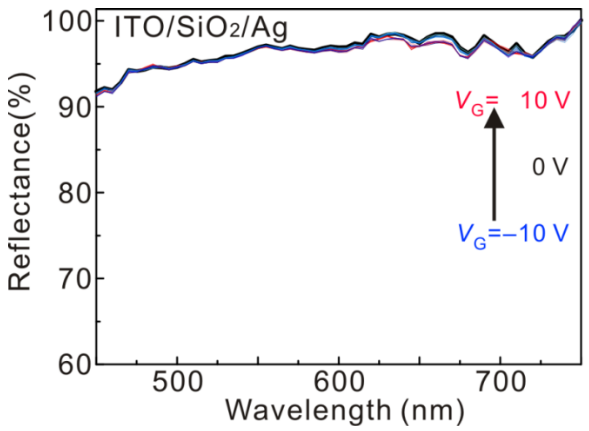

Supplementary Figure 18 | Reflectance spectrum of indium tin oxide (ITO)/SiO_2/Ag gated heterostructure. No significant reflectance modulation is observed when an applied bias is varied between –10 V and +10 V. Since the thickness of the SiO_2 gate dielectric is 9 nm, the maximal strength of the generated DC electric field is 11 $MVcm^{-1}$. The carrier concentration of the deposited 10 nm-thick ITO film is 3×10^{20} cm^{-3}, and the epsilon-near-zero (ENZ) wavelength of the ITO film is located in the near-infrared wavelength range (around telecom wavelength). As a result, ITO transitions from optically dielectric to optically metallic phase in the near-infrared wavelength range. Since this transition is crucial for the observation of optical modulation, the ITO/SiO_2/Ag gated heterostructure does not show significant optical modulation in the visible wavelength range.

Supplementary References

1 Fillot, F. *et al*. Investigations of titanium nitride as metal gate material, elaborated by metal organic atomic layer deposition using TDMAT and NH_3. *Microelectron Eng.* **82**, 248–253 (2005).

2 Patsalas, P., Kalfagiannis, N. & Kassavetis, S. Optical properties and plasmonic performance of titanium nitride. *Materials* **8**, 3128–3154 (2015).

3 Braic, L. *et al*. Titanium oxynitride thin films with tunable double epsilon-near-zero behavior for nanophotonic applications. *ACS Appl. Mater. & Inter.* **9**, 29857–29862 (2017).

4 Logothetidis, S., Alexandrou, I. & Papadopoulos, A. In situ spectroscopic ellipsometry to monitor the process of TiN_x thin films deposited by reactive sputtering. *J. Appl. Phys.* **77**, 1043–1047 (1995).

5 Zgrabik, C. M. & Hu, E. L. Optimization of sputtered titanium nitride as a tunable metal for plasmonic applications. *Opt. Mater. Express* **5**, 2786–2797 (2015).

6 Wang, Y., Capretti, A. & Dal Negro, L. Wide tuning of the optical and structural properties of alternative plasmonic materials. *Opt. Mater. Express* **5**, 2415–2430 (2015).

7 Huang, Y. W. *et al*. Gate-tunable conducting oxide metasurfaces. *Nano Lett.* **16**, 5319–5325 (2016).

8 Novotny, L. & Hecht, B. *Principles of nano-optics*. 2nd edn. (Cambridge University Press, 2012).

9 Hoang, T. B., Akselrod, G. M. & Mikkelsen, M. H. Ultrafast room-temperature single photon emission from quantum dots coupled to plasmonic nanocavities. *Nano Lett.* **16**, 270–275 (2016).

10 Akselrod, G. M. *et al*. Probing the mechanisms of large Purcell enhancement in plasmonic nanoantennas. *Nat. Photon.* **8**, 835–840 (2014).

11 Kubin, R. F. & Fletcher, A. N. Fluorescence quantum yields of some rhodamine dyes. *J. Lumin.* **27**, 455–462 (1982).

12 Hoang, T. B. *et al*. Ultrafast spontaneous emission source using plasmonic nanoantennas. *Nat. Commun.* **6**, 7788 (2015).

13 Thyagarajan, K., Sokhoyan, R., Zornberg, L. & Atwater, H. A. Millivolt modulation of plasmonic metasurface optical response via ionic conductance. *Adv. Mater.* **29**, 1701044 (2017).

14 Emboras, A. *et al*. Atomic scale plasmonic switch. *Nano Lett.* **16**, 709–714 (2016).

15 Hoessbacher, C. *et al*. The plasmonic memristor: A latching optical switch. *Optica* **1**, 198–202 (2014).
